# Hereditary Angioedema: A Gynecology and Obstetrics Perspective

**DOI:** 10.7759/cureus.19861

**Published:** 2021-11-24

**Authors:** Francisco Évora, Ana Rodolfo

**Affiliations:** 1 Obstetrics and Gynecology, Coimbra Hospital and University Center, Coimbra, PRT; 2 Allergy and Immunology, Hospital da Horta, Horta, PRT

**Keywords:** prophylaxis, attacks treatment, c1 inhibitor, icatibant, laryngeal edema, abdominal pain, triggers, bradykinin, non-histaminergic angioedema, hereditary angioedema

## Abstract

Hereditary angioedema is an autosomal dominant genetic disease that causes tissue edema mediated by bradykinin. The angioedema attacks have several triggers including stress, trauma, infection, and increased estrogens levels. This explains the greater incidence and clinical severity in women, which are usually asymptomatic until puberty, when the attacks begin to occur. It may involve multiple locations on the body, leading to complications, such as surgical intervention prompt by severe acute abdominal pain, and laryngeal edema that can culminate in death from asphyxia. This is of particular concern as this angioedema does not respond to life-saving medications commonly used in its treatment, namely, high doses of second-generation antihistamines, corticosteroids, and epinephrine. Hereditary angioedema attacks are treated with specific medication that includes icatibant, ecallantide, and C1 inhibitor, the latter being also used in short-term and long-term prophylaxis. There are other pharmacological strategies for long-term prophylaxis like lanadelumab, danazol, stanozolol, aminocaproic acid, and tranexamic acid. During pregnancy and lactation, the preferred treatment and prophylaxis is C1 inhibitor.

We report a case of hereditary angioedema describing its chronological evolution over a period of a woman's life, and highlighting some of the specificities of this pathology that intersect with the specialty of Obstetrics and Gynecology. Our aim is to draw attention to these particularities, namely the triggering factors of crisis, the need for high suspicion of the diagnosis, and the treatment and prophylaxis options for pregnant and non-pregnant women that can make the difference between life and death. To achieve a favorable outcome, the multidisciplinary teamwork between the specialties of Immunoallergology and Obstetrics and Gynecology was crucial.

## Introduction

Hereditary angioedema (HAE) is a rare (1 in 50.000) but potentially life-threatening disorder characterized by attacks of cutaneous and submucosal swelling [1]. It is an autosomal dominant genetic disease, with a family history in 75% of cases, the remaining 25% corresponding to de novo mutations [2].

HAE is classified into two large groups: HAE with a deficit of C1 inhibitor, which can be quantitative (type 1) or functional (type 2); and HAE with normal C1 inhibitor (previously called type 3), which can originate from mutations of several genes such as coagulation factor XII, plasminogen, angiopoietin-1 or kininogen-1 heavy chain [2-4].

In pathophysiological terms, HAE results from an increase in bradykinin with consequent vasodilation and increased vascular permeability, which clinically translates into angioedema with non-histaminergic characteristics [1].

The objective of this clinical case is to alert Obstetrics and Gynecology specialists to the specificities of diagnosis and treatment of this pathology, since hormonal influences of pregnancy or exogenous estrogens may be pronounced, particularly in patients who have HAE with a factor XII mutation [1].

## Case presentation

An 18-year-old nullipara was referred for a gynecology consultation due to multiple episodes of severe and acute abdominal pain that interfered with activities of daily living. At 16 years of age, she underwent appendectomy due to intense abdominal pain although the appendix showed no macroscopic or microscopic changes. She had no other relevant personal history. She was only medicated with oral estroprogestative (ethinylestradiol 0.02 mg + gestodene 0.075 mg). In the gynecology consultation, a laboratory and pelvic echographic study was carried out, which showed no changes. The patient was even inconclusively evaluated by other specialties, maintaining the abdominal pain crisis.

At 23 years of age, she begins having episodes of facial edema, sometimes accompanied by oropharyngeal edema, in the context of respiratory infections or after dental procedures. She went to the ER multiple times, having been medicated with intravenous antihistamines and corticosteroids, but with poor response to this therapy, with symptomatic resolution occurring only after two to five days.

At the age of 26 years, she had an episode of angioedema after taking an oral medication (bacterial lysate for the prophylaxis of respiratory infections) and was referred to an immunoallergology consultation for suspected drug allergy. In the immunoallergology consultation, a family history of similar symptoms was identified in one sister, two paternal aunts, and one paternal cousin. In view of the clinical manifestation of disfiguring and non-pitting angioedema with non-histaminergic characteristics (asymmetric, non-pruritic, without urticaria, with no response to antihistamines or corticosteroids), severe abdominal symptoms, and the presence of a family history of similar symptoms, the diagnostic hypothesis of HAE was raised. In this context, a laboratory study was requested that included the complement cascade, namely the quantitative assay of C1 inhibitor (0.21 g/L, normal range 0.21 to 0.39 g/L), the activity of C1 inhibitor (77%, normal range >40%), and C4 (0.12 g/L, normal range 0.10 to 0.40 g/L), which were all within normal values. Given the high clinical suspicion, we requested the search for the genetic mutation of coagulation factor XII. The pathogenic variant Thr309Lys was identified in exon 9 of the gene, which confirmed the existence of HAE with mutation in coagulation factor XII. Hence, a gynecology consultation was requested to replace estroprogestative contraception with an estrogen-free method, as well as a genetics consultation for an eventual genetic study of family members up to the third degree of kinship. It was recommended short-term prophylaxis with a C1 inhibitor concentrate, prior to invasive procedures. The patient was advised to always bear clinical information containing the diagnosis and treatment to be administered in the event of a crisis. After stopping contraception with estrogens, she remained asymptomatic until 28 years of age, when she first got pregnant.

She began follow-up in the obstetrics department, with support from the immunoallergology department, presenting during pregnancy three crisis of angioedema, one on the foot and two on the face. The first attack occurred at 20 weeks of pregnancy when the patient had unilateral edema of the right foot that spontaneously resolved in two days. At 21 weeks she had another attack with edema of the lower half of the face, including the lips. As the edema was getting progressively more severe, the patient went to the ER. She was treated with 1500 UI of C1 inhibitor concentrate and was asymptomatic after about four hours. At 24 weeks she presented to the ER with another episode of edema affecting the left part of the face, including the left part of the lips. She was again treated with C1 inhibitor concentrate. For labor, a prophylaxis plan was drawn up together with the immunoallergology department, consisting of the administration of 1500 IU of C1 inhibitor approximately 2 hours before delivery. She was admitted to obstetrics due to spontaneous rupture of membranes and the delivery occurred after 10 hours by vacuum extraction, being born a boy weighing 3595 grams and with an Appearance, Pulse, Grimace, Activity, and Respiration (APGAR) score of 9/10/10. There were no complications during the puerperium. At the six-week postpartum consultation, it was decided to maintain contraception with an oral isolated progestative (desogestrel 0.075 mg).

## Discussion

The mechanism of swelling in HAE is not yet fully characterized but involves enhanced bradykinin signaling [5]. The most common cause of HAE with normal C1 inhibitor levels is the factor XII gene mutation p.Thr309Lys, which was found to cause a reduced threshold for activation of the contact/kinin system [6]. HAE is an autosomal dominant condition with incomplete penetrance, particularly affecting females (males are frequently silent carriers) [7].

HAE attacks can have several triggers including stress, trauma, infection, and increased concentration of estrogens of endogenous (pregnancy, puberty, and menstrual cycle) or exogenous (contraception and hormonal therapy) origins. HAE with factor XII mutation is particularly triggered by estrogens [8]. For this reason, HAE is a contraindication for estrogens, and hormonal contraception must be carried out with isolated progestative. This is why gynecologists and obstetricians can meet these patients in their practice, as two of the main triggers for attacks are estrogen-containing therapies and pregnancy [2,8].

The most important step in making a diagnosis of HAE is to maintain a high index of suspicion. The diagnosis should be made as soon as possible, so that the patient may receive adequate treatment, particularly during severe attacks. Patients are usually asymptomatic until puberty [9]. Clinical features include recurrent cutaneous and submucosal angioedema without urticaria or pruritus. The most frequent location of angioedema is the face (usually mouth, eyelids, and ears), followed by neck (pharynx and larynx), extremities, and gastrointestinal (abdominal pain) [1]. It must be taken into account that all HAE patients are at risk for laryngeal edema, and more than 50% have a laryngeal attack during their lifetime, that will not respond to antihistamines, corticosteroids, or epinephrine [10,11]. This laryngeal edema can be fatal, leading to death from asphyxiation. The gastrointestinal wall edema can cause severe abdominal pain and be diagnosed as a case of surgical acute abdominal pain, leading to unnecessary exploratory laparotomies/laparoscopies, appendectomies, or other invasive procedures. There is literature showing that one-third of patients experiencing abdominal symptoms underwent appendectomies and exploratory laparotomies [12]. This is possibly what happened in this clinical case, as the laparotomy did not show pathological changes and the histological analysis of the removed appendix was normal.

Once a history of recurrent angioedema with non-histaminergic characteristics is identified (including lack of response to the previously mentioned treatments), and in the absence of medications known to cause angioedema (as angiotensin-converting enzyme inhibitors), family history of similar episodes should be investigated [1,11]. A laboratory study of the complement cascade must follow, which includes C4 and C1 inhibitor antigen levels as well as C1 inhibitor function. In the case of HAE with normal C1 inhibitor levels, all of the above will be within normal ranges [9]. In this case, a genetic study will be needed to identify a factor XII mutation. Other mutations can also be identified, such as mutation on angiopoietin-1, plasminogen, or kininogen-1; other mutations are being identified as causes for HAE with normal C1 inhibitor levels [6].

The treatment of HAE with factor XII mutation is similar to that of HAE with C1 inhibitor deficiency [9]. The main point for successful treatment is to understand the physiopathology of HAE. Thus, it is essential to remember that angioedema is mediated by bradykinin [5]. The treatment has three components: treatment for acute attacks/crisis, short-term prophylaxis, and long-term prophylaxis.

According to the most recent guidelines, all HAE attacks are eligible for treatment, regardless of the location of the swelling or the severity of the attack [13]. Approved acute treatments include icatibant (FIRAZYR®, Takeda), ecallantide (KALBITOR®, Takeda), plasma-derived C1 inhibitor (BERINERT®, CSL Behring), and recombinant human C1 inhibitor (RUCONEST®, Pharming) [9]. Given the pathophysiology of HAE, the preferred acute treatment will be icatibant, which is a bradykinin B2 receptor antagonist [5]. However, icatibant and ecallantide should not be used during pregnancy and lactation [9]. In these cases, the preferred acute treatment is C1 inhibitor [8].

Short-term prophylaxis may be indicated before invasive procedures [1], which are known triggers as discussed before. These may include labor, amniocentesis or chorionic villus sampling, application of intrauterine devices, and surgeries [8]. The preferred prophylaxis is a C1 inhibitor, administered one to 12 hours (preferably within two hours) before the procedure. If the C1 inhibitor is unavailable, fresh-frozen plasma (two units administered one to 12 hours before the procedure) can be used [1]. Therefore, it is recommended to have a prepartum evaluation, to program the administration of short-term prophylaxis before labor, and to ensure that on-demand treatment is available in the case of an attack.

Long-term prophylaxis should be considered for patients who have frequent or severe attacks and can include 17α-alkylated androgens (such as danazol and stanozolol) and antifibrinolytic agents (aminocaproic acid and tranexamic acid) [9]. More recently a monoclonal antibody inhibitor of plasma kallikrein (lanadelumab) was approved at a dose of 300 mg every two weeks [14]. During pregnancy and lactation, the only options for long-term prophylaxis are tranexamic acid (except on the first trimester) and C1 inhibitor [9].

## Conclusions

Although HAE is a rare condition, it is potentially fatal. Therefore, proper planning of hormonal therapy and invasive procedures in these patients is vital. In this sense, it is important that obstetricians and gynecologists have an idea about this pathology so that it can be managed in the best way in collaboration with an immunoallergologist.

All HAE attacks are eligible for treatment, regardless of the location of the swelling or the severity of the attack. This is due to the fact that all HAE patients are at risk of life-threatening laryngeal edema that will not respond to antihistamines, corticosteroids, or epinephrine. The timely treatment of the crisis is essential, as well as prophylaxis measures to prevent its occurrence.

## References

[1] Busse PJ, Christiansen SC (2020). Hereditary Angioedema. N Engl J Med.

[2] Patel G, Pongracic JA (2019). Hereditary and acquired angioedema. Allergy Asthma Proc.

[3] Bork K, Wulff K, Steinmüller-Magin L, Braenne I, Staubach-Renz P, Witzke G, Hardt J (2018). Hereditary angioedema with a mutation in the plasminogen gene. Allergy.

[4] Bafunno V, Firinu D, D'Apolito M (2018). Mutation of the angiopoietin-1 gene (ANGPT1) associates with a new type of hereditary angioedema. J Allergy Clin Immunol.

[5] Cicardi M, Zuraw BL (2018). Angioedema due to bradykinin dysregulation. J Allergy Clin Immunol Pract.

[6] Veronez CL, Csuka D, Sheikh FR, Zuraw BL, Farkas H, Bork K (2021). The expanding spectrum of mutations in hereditary angioedema. J Allergy Clin Immunol Pract.

[7] Bova M, Suffritti C, Bafunno V (2020). Impaired control of the contact system in hereditary angioedema with normal C1-inhibitor. Allergy.

[8] Yakaboski E, Motazedi T, Banerji A (2020). Hereditary angioedema: special considerations in women. Allergy Asthma Proc.

[9] Zuraw BL (2008). Clinical practice. Hereditary angioedema. N Engl J Med.

[10] Bork K, Hardt J, Witzke G (2012). Fatal laryngeal attacks and mortality in hereditary angioedema due to C1-INH deficiency. J Allergy Clin Immunol.

[11] Lumry WR (2013). Overview of epidemiology, pathophysiology, and disease progression in hereditary angioedema. Am J Manag Care.

[12] Patel N, Suarez LD, Kapur S, Bielory L (2015). Hereditary angioedema and gastrointestinal complications: an extensive review of the literature. Case Reports Immunol.

[13] Busse PJ, Christiansen SC, Riedl MA (2021). US HAEA Medical Advisory Board 2020 guidelines for the management of hereditary angioedema. J Allergy Clin Immunol Pract.

[14] Banerji A, Riedl MA, Bernstein JA (2018). Effect of lanadelumab compared with placebo on prevention of hereditary angioedema attacks: a randomized clinical trial. JAMA.

